# Does Parental Migration Have Negative Impact on the Growth of Left-Behind Children?—New Evidence from Longitudinal Data in Rural China

**DOI:** 10.3390/ijerph14111308

**Published:** 2017-10-27

**Authors:** Xu Tian, Caicui Ding, Chong Shen, Hui Wang

**Affiliations:** 1 College of Economics and Management, China Center for Food Security Studies, Nanjing Agricultural University, Nanjing 210095, China; xutian@njau.edu.cn; 2 Department of Nutrition and Health Education, National Institute for Nutrition and Health, Chinese Center for Disease Control and Prevention, Beijing 102206, China; kacyting@163.com; 3 Department of Epidemiology and Biostatistics, School of Public Health, Nanjing Medical University, Jiangning District, Nanjing 211166, China; sc@njmu.edu.cn

**Keywords:** left-behind children, height, weight, calorie, carbohydrate

## Abstract

The soaring number of left-behind children (LBC) in China has raised concerns about whether or not they can receive adequate care. This study investigated the impact of parents’ migration on LBC’s growth. LBC were divided into father-left children (F-LBC) and at least mother left children (M-LBC), both of which were compared with non-left-behind children (non-LBC) in terms of growth indicators. Data of 466 children with two continuous measurements were obtained from the four recent waves of the China Health and Nutrition Survey (CHNS). Anthropometric measures and 24-h recall of three consecutive days of dietary intake were extracted. The disparity of growth and nutrition status were compared by the difference-in-difference (DID) method. Results showed that LBC had significantly worse height and weight than non-LBC at baseline, respectively (*p* = 0.006, *p* = 0.003). This disadvantage was improved after parental migration, especially for M-LBC. However, the impact on growth status caused by parents’ migration was statistically insignificant once the pre-treatment disparity was removed. Further analysis on nutrition status indicated that fathers’ migration had a significant negative impact on F-LBC’s calorie intake (*p* = 0.014), which was mainly caused by the decline of carbohydrates (*p* = 0.008). This study indicated that the negative impact detected in previous studies might be caused by the retarded growth of LBC before parents’ migration.

## 1. Introduction

China has been undergoing rapid development and urbanization in the past four decades, which has caused large number of rural residents, particularly young adults aged 25–49 years old, to migrate from their homes in rural areas to cities in search of better job opportunities [1,2,3,4,5,6]. The National Statistic Bureau of China (NSBC) claimed that the total number of migrant workers had reached 277 million in 2015 [7]. Due to financial constraints and restrictions on migrants’ access to education and health services in urban areas, children in migrant families are left behind in their hometowns with a caregiver, usually the paternal grandparents [8,9]. Those children are termed as “left-behind children (LBC)” [3]. A report released in 2013 announced that the total number of LBC in rural China was 61.0 million, which accounts for 37.7% of the total number of children in rural areas and 21.9% of the total number of children in China [4,8,10].

Previous studies in various countries have shown that migrant families have adverse impacts on healthy development among children [3,9,11]. Parental migration was found to be associated with less physical activity of school-aged children in Mexico [12]; reduced health outcomes in India, Peru, and Vietnam; decreased cognitive ability on test scores in India and Vietnam [13]; and higher risks of being stunted in middle childhood for Vietnamese children during the first 36 months [14]. Several studies in China also presented strong evidence of the negative association between parental migration and LBC’s development, particularly in terms of mental health, physical health, nutrition, and education [8,15]. For instance, LBC were found to have slower height growth and weight gains in rural China [11], less academic assistance and adequate supervision at home in rural areas [16], less protein intake and higher fat intake for boys [17], higher risk of unhealthy diet (such as hating vegetables and fruit) and physical inactivity [3,9,18], and deficient intake of most micronutrients [4]. As a response to the perception that LBC are more vulnerable than non-left-behind children (non-LBC), the Chinese government developed a number of programs, such as supplemental education support, uptake of childhood vaccination, emotional support, and community-based child protection services, to give preferential treatment or assistance in social services to LBC [8].

The majority of current studies, however, only compare LBC and non-LBC using cross-sectional data. As we know, with cross sectional data it can be very difficult to identify the causality between two variables. Particularly, if the migration decision of parents is correlated with children’s health, nutrition, and education status, the estimation based on cross sectional data might be biased. Only several studies adopted longitudinal data to investigate the impact of parental migration on children’s development. One study [11] compared the height and weight growth of LBC and non-LBC at different life stages using a growth curve model. They found that boys left behind in early childhood have slower height and weight growth than their counterparts. Another two studies [4,17] both found some evidence of deficient intake of protein for LBC compared with non-LBC. On the contrary, a recent study [8] re-examined the issue by pulling data from a comprehensive data set covering 141,000 children in ten Chinese provinces between 2009 and 2013. They found that LBC performed as well as or even better than non-LBC in terms of health, nutrition, and education. In conclusion, studies using longitudinal data found more conservative results as compared with those using cross sectional data. We thus revisit the topic by deriving data from the China Health and Nutrition Survey (CHNS). In particular, we focus on the growth status of LBC and non-LBC. Moreover, in order to avoid the bias caused by unobservable heterogeneity between LBC and non-LBC before migration of parents, we adopted a difference-in-difference (DID) method to identify the average treatment effect of treated (ATT) of parental migration on children’s growth. In addition, a kernel-based propensity score matching (PSM) was combined with DID to test the robustness of the results. This study contributes to the current literature in two ways: first, using a longitudinal data can better identify the causality relationship between parents’ migration and the health and nutritional status of left-behind children; second, DID and PSM-DID provide a way to remove the pre-treatment heterogeneity, which could increase the accuracy of estimation.

## 2. Materials and Methods

### 2.1. Study Design and Participants

Participants in our study were drawn from the recent four waves (2004, 2006, 2009, and 2011) of the CHNS. The CHNS is an ongoing open-cohort study, which consists of data obtained from about 4400 households employing a multi-stage, random cluster strategy for nine provinces (Liaoning, Heilongjiang, Jiangsu, Shandong, Henan, Hubei, Hunan, Guangxi, and Guizhou). Three mega cities, Beijing, Chongqing, and Shanghai, joined the survey in 2011. The survey design and methods are described in detail elsewhere [18]. The survey was approved by the institutional review committees from the University of North Carolina at Chapel Hill and the National Institute for Nutrition and Food Safety, China Center for Disease Control and Prevention. All participants (or their parents/guardians) provided written informed consent for their participation in the survey. Moreover, the study followed all the relevant ethical regulations.

LBC were defined as those with one or both parents left home to work/study elsewhere at the time of survey. We focused on children younger than 18 years old (*n* = 3078). After combining nutrition data with other datasets such as physical activity, 158 observations were lost due to incomplete information (*n* = 2820). Only children with more than two observations were included (*n* = 1840). Furthermore, to form strongly balanced panel data, we only kept children with exactly two period observations: children whose parents stayed with them at the first survey time, while their parents migrated before the second survey time were defined as LBC (*n* = 149), and those whose parents lived with them at both survey times were defined as non-LBC (*n* = 480). After merging other information (family characteristics, socio-economic factors), 366 observations (183 children) were removed due to incomplete information. Finally, 446 children (each of which had two observations) remained in our analysis, of which 346 are non-LBC, and 100 are LBC. We further categorized LBC into two groups: fathers migrated only (F-LBC) and at least mother migrated with or without a migrant father (M-LBC). The numbers of children for these two groups were 62 and 38, respectively. The distribution of observations over four waves were presented in Table S1 in the Supplementary File. To check the representativeness of selected samples, we also presented the comparison of characteristics of selected and excluded samples in terms of mean and standard deviation. Even though some characteristics are significantly different between selected and excluded groups, the magnitude of these differences is negligible (see Table S2 in the Supplementary File). We thus conclude that our sample is representative.

### 2.2. Growth Indices

For each child, anthropometric measurements (including height/length and weight) were measured by trained health workers using regularly calibrated equipment according to the manufacturer’s instructions (Seca Digital Floor Scale (SECA) 880 scales and SECA 206 wall-mounted metal tape). Body Mass Index (BMI) was calculated by dividing the weight (kg) by the square of the height (m^2^) of each participant. Furthermore, children’s growth indicators were strongly correlated with age and sex; direct comparison between LBC and non-LBC were meaningless if these two groups were not equally distributed across age and gender. Therefore, the Chinese Children Growth Standards, a growth reference for various ages and sexes based on a nine-city pilot study in China, was adopted to remove the impact of age and sex [19] (please see Table S3 in the Supplementary File). We did not use the World Health Organization (WHO) growth standards because Chinese children were not included in estimating the basis of the WHO standards, and the growth curve of Chinese adolescents varied from that of other countries [20]. In addition, the WHO standards only provide weight references for children younger than 11 years old, but our sample included children aged between 11 and 18 years old.

### 2.3. Nutrition Indices

Dietary data were collected using 24-h recall method by trained field interviewers. Interviewers recorded the type and amount of all food consumed at and away from home during the 24-h period of the previous day with the help of food models and picture aids. Food consumption of children younger than 12 years was measured by asking the caregiver who prepared the food in the household. Detailed information about collecting dietary data can be found elsewhere [18]. To further calculate macronutrient intake, we adopted the China Food Composition [21] to convert the detailed food consumption data into intake of calorie, protein, fat, and carbohydrate. To remove the food waste during processing, the edible proportion of each food item was also used in the conversion. Finally, the referenced nutrient intake proposed in the Chinese Dietary Reference Intake Handbook was adopted to normalize the real intake of all nutrients. After normalization, nutrition intake could be compared directly, regardless of age and sex [20].

### 2.4. Other Covariates

Household characteristics such as household net income, household size, ratio of children in the household, characteristics of children (age and gender), and household head (age, gender, education level, and physical activity) were also collected during the survey. The time gap between the baseline and follow-up periods was calculated as well. These covariates were used in our analysis to match LBC and non-LBC.

### 2.5. Statistical Analysis

Children living with both parents (non-LBC) were set as the reference group for statistical comparison. Descriptive statistics were presented as means ± SD. To compare the growth and nutrition indicators of LBC and non-LBC, *t* tests were applied. In addition, the Holm–Bonferroni correction of the *p* values was adopted in multiple comparisons (F-LBC and M-LBC against the non-LBC).

The impact of parental migration on children’s growth and nutrition status was measured by the average treatment effect of the treated group (ATT), which is estimated by the DID method. The covariates used in estimation including the aforementioned characteristics of household, children, and household head. In addition, the time gap between the baseline and follow-up periods might also affect the impact of parental migration on the outcomes. We thus also included it as a covariate in the estimation. For a robustness check, a kernel-based propensity score matching (PSM) DID is also conducted.

All statistical analyses were conducted using Stata version 14 (StataCorp, College Station, TX, USA), and the statistical significance level was set at *p* < 0.05 (two-sided).

## 3. Results

### 3.1. Characteristics of the Participant Groups

Table 1 reported the comparison of characteristics of household, household head, and children for different families. Heterogeneity was found in most indices between families with no migrants and those with migrants. For instance, families with migrated mother had significantly lower per capita income than families without any migrants (*p* < 0.01). In addition, the household size, characteristics of household head (activity level, sexes, education attainment) with no migrants and those with migrants were also significantly different (all *p* < 0.05). In addition, the average time gap between the baseline and follow-up periods were 3.0–3.2 years, and no significant difference was detected between LBC and non-LBC.

### 3.2. Comparison of Growth Indices for Different Children

Table 2 reported the growth indices of children from different families. The upper section presented the absolute values of growth indices of children, and the lower section showed the normalized values adjusted by age and sex. Values in both periods were reported in each section: the first period (baseline) and the second period (follow-up). In the follow-up period of absolute value, LBC had significantly lighter weight than non-LBC (*p* < 0.01). In particular, M-LBC had smaller BMI than non-LBC due to relatively light weight (*p* < 0.01). However, if we traced all children back to the year before parents’ migration (the baseline period), we found that LBC already had retarded growth compared to non-LBC (all *p* < 0.01) by using the normalized value. This phenomenon was improved after parents’ migration in normalized values, especially for M-LBC. The BMI of M-LBC was still significant lower in comparison with non-LBC due to relatively lighter weight (*p* < 0.01).

### 3.3. The ATT of Parents’ Migration on Children’s Growth

In order to confirm the effect of migration on the disparity of growth indices, the DID method, which adjusted the characteristics of the family (income, size, proportion of children), and household head (age, activity, gender, education), was implemented. Results were presented in Table 3. After removing the effect of social-economic factors, the LBC was significantly lighter and shorter than non-LBC at the baseline, respectively (*p* = 0.006, *p* = 0.003), especially for M-LBC (*p* = 0.005, *p* = 0.009). After migration, the difference in height and weight between M-LBC and non-LBC decreased, but the BMI of M-LBC was smaller than that of non-LBC (*p* = 0.011) due to relatively smaller weight of M-LBC (*p* = 0.031).

Intriguingly, F-LBC was statistically shorter than non-LBC at the baseline (*p* = 0.034), and the difference remained after migration (*p* = 0.013). In general, the weight and height gap between non-LBC and LBC narrowed down in the follow-up period, particularly for children whose mother migrated. However, the overall results, measured by the ATT, indicated that migration of parents had no significant impact on LBC’s growth after removing the pre-treatment heterogeneity. Similar results were also found using the PSM-DID method (see Table S4 in the Supplementary File).

### 3.4. The Differences of Dietary Patterns among Different Children

After adjusting all social-economical status, the F-LBC had significantly higher intake of carbohydrate than non-LBC at the baseline (see Table 4). During the follow-up period, the normalized nutrition indices dropped down for all children (differently, the carbohydrate intake of non-LBC increased slightly). In particular, children whose father migrated experienced largest decline in protein and carbohydrate (DID was −0.112 and −0.394, respectively). Consequently, the total energy intake declined significantly, reflected by the significant ATT (*p* = 0.014). In addition, M-LBC had significantly lower fat intake compared with non-LBC in the follow-up period (*p* = 0.021). Same results were also detected using the PSM-DID method (see Table S5 in the Supplementary File).

## 4. Discussion

The notable finding of the present study was that the LBC had the retarded growth status before their parents migrated to urban area in comparison with their counterpart. This gap narrowed down slightly after the migration of LBC parents. However, the disparity was still significant by directed comparison in the follow-up period. After removing the pre-treatment heterogeneity using the DID method, no significant association between migration and growth status of LBC was detected. To illustrate the plausibility of insignificant difference in growth status, further analysis on the macro-nutrients intake was performed. Results indicated that all rural children suffered from insufficient energy intake, which could be attributed to inadequate fat intake. On the contrary, all children except for the M-LBC had adequate protein intake, and the intake of carbohydrate was much higher than the referents for all rural children. Intriguingly, the intake of carbohydrate significantly declined after father migrated to urban area. In total, migration of father had significant negative impact on children’s intake of carbohydrate, and further resulted in lower energy intake for F-LBC. However, migration of mother had no significant impact on the nutrition intake of M-LBC. 

In our analysis, the growth status of LBC was retarded at the baseline and slightly improved after their parents migrated to urban area. This result contradicted with previous studies. Several cross-sectional studies indicated that LBC in rural China were more vulnerable to retarded growth (i.e., stunting and underweight). However, those cross-sectional studies did not remove the growth and nutrition disparity between LBC and non-LBC in the baseline period [22,23,24]. Thus their results might be biased due to the pre-treatment heterogeneity. Although one longitudinal analysis (1997–2009) suggested that boys who were left behind were restricted in height and weight compared with their counterparts [11]. However, the author did not provide the baseline information about the growth status and the migration period, which might bias the final results to a certain degree [11]. Additionally, one cross-sectional study, which was carried out in Guangdong, did not find significant differences between LBC and non-LBC, and LBC were even more likely to be taller and heavier than their peers [3]. Another study reviewed 27 surveys conducted between 2009 and 2013 and indicated that LBC performed as well as or better than children living with both parents in terms of health. One study focused on 0–3 year-old children adopting the UNICEF China’s maternal and children health survey data, the author indicated that left behind status was not associated with stunting [9], even though the first 1000 days were critical for children’s growth [25,26]. The study claimed that the educational attainments of parents were positively associated with the height of children, which was consistent with our analysis. We did find that the educational level of LBC’s parents was generally lower compared with non-LBC’s parents.

Intriguingly, both standardized height and weight of M-LBC increased after mothers’ migration, and the gap between M-LBC and non-LBC narrowed down. This finding contradicted with most published documents, which claimed that mothers was likely to consume high quality foods in the absence of migrant fathers, thus the nutrition status of M-LBC should deteriorate and growth should also be negatively affected once the mother migrated [27]. However, in rural China, fathers generally are the main labor force in the household in rural region, and the “best food” was traditionally kept for them. When father migrated to urban region, the housewife spontaneously reduced the consumption of high-quality foods. Thus, the carbohydrate and total energy intake declined consequentially. In addition, women generally dominated the kitchen in China. Once the mother migrated to urban region while father stayed at home, M-LBC’s nutrition status would depend on father’s dietary preference. As men usually preferred more animal food, the gap of protein and fat intake between M-LBC and non-LBC decreased. Accordingly, the gap of height and weight between M-LBC and non-LBC also narrowed down, but the change was statistically insignificant. The possible reason was that the observed migration duration was very short (around three years), while the impact of nutrition on growth needed a longer time to show up. Hence, the adverse effects of migration on growth status might be underestimated.

Overall, the present analysis indicated that parents’ migration did not have significant impact on children’s growth status. However, we did find that LBC’s disadvantages in height and weight were alleviated slightly after parents’ migration, which is consistent with study of Zhou et.al. [8]. One reason for this phenomenon might be the “care versus resources” trade off. LBC receive less face-to-face care from their parents than their peers. However, they might get more financial support from their parents who are working in big cities. Another explanation might be that parents who migrated to urban areas and leave their children with other guardian may be different from those who decided to stay at home. For instance, young couples were more likely to migrate if the guardian candidates were capable of taking care of children. Therefore, the LBC can be still taken care of in a good way and receive more household resources. Our finding indicated that the negative impact of lack of direct care caused by parental migration might be offset by the remittance sent back home. Therefore, more attention should be paid to the psychological health rather than physical health. Because the latter can be easily offset by increased family income, while the former one is more difficult to cure. Policy makers aimed at helping left-behind children should think about providing more psychological assistance and counseling in rural areas, especially in primary and middle schools where left-behind children spend most of their time.

There are several limitations in our analysis. First, restricted by the definition of LBC and the design of the present study, the sample size of LBC was relatively small. The number of both-parent-left children was four, hence that group was combined with children whose mothers migrated to urban areas, which might underestimate the adverse effect of mothers’ migration. Future research with larger sample sizes should be conducted to increase the effectiveness of statistics. Second, previous studies claimed that the remittance and frequency of long-distance communication can improve the nutritional status and health status of LBC, which were not included as co-variants in the present study due to data limitations. Third, the intake of snacks was not counted due to missing data, which might lead to underestimation of LBC’s nutrition intake.

Despite the aforementioned disadvantages, to our knowledge, the present study is the first study using the DID method and combining the nutritional status and growth status in one analysis. After removing the pre-treatment disparity, our study provided a more precise estimation of the effects of parents’ migration. In addition, this study distinguished migration of father from mother, and found that migration of the mother could reduce the gap between M-LBC and non-LBC, while migration of the father had no such impact. Our study indicated that parents’ migration does not always have a negative impact on LBC’s growth and nutrition status, and the negative impact detected in previous studies might be caused by the disparity before parents’ migration. Moreover, several studies indicated that the nutritional knowledge and food preferences of left behind children’s caregivers can contribute to children’s eating habits [28]. Although under-nutrition was primarily caused by nutrient deficiency, which is based on the adverse economic status, evidence represented that most under-nutrition among children was largely attributable to a lack of knowledge with respect to healthy diet patterns rather than food shortage [14,29]. Therefore, LBC’s diets might be more likely to be affected by their care givers, thus nutrition education for people who are taking care of LBC could be a more effective way to reduce the negative impact of migration.

## 5. Conclusions

This study reexamined the impact of parents’ migration on the growth indicator of LBC. We found that LBC were significantly shorter and lighter than non-LBC at the baseline period, and this disadvantage was slightly improved after parental migration, especially for M-LBC. However, after removing the pre-treatment heterogeneity between LBC and non-LBC using a DID method, no significant impacts of parents’ migration on LBC’s height and weight was detected. Our study indicated that growth retardation of LBC detected in previous studies might be caused by the growth disparity between LBC and non-LBC before parent’s migration. In other words, growth retardation of children could be the cause—rather than the effect—of parental migration.

## Figures and Tables

**Table 1:** Comparison of characteristics between different families.

Variable	Non-Left (*n* = 692)	Left-Behind (*n* = 200)	Father-Left (*n* = 124)	At Least Mother-Left (*n* = 76)
*Household characteristics*				
Income (Yuan/year)	28,812 ± 35,726 **^a^**	26,175 ± 27,932	29,460 ± 32,682	20,815 ± 16,479 *
Income (median)	20,272	17,407	18,140	17,227
Household Size	4.7 ± 2.0	5.1 ± 2.0 *	5.3 ± 2.2 *	4.8 ± 1.6
Child ratio **^b^**	0.4 ± 0.1	0.4 ± 0.1	0.4 ± 0.1	0.4 ± 0.2
*Household head’s characteristics*				
Age (Year)	44.8 ± 12.6	44.9 ± 12.7	45.0 ± 13.0	44.6 ± 12.2
Activity	3.1 ± 1.1	3.4 ± 1.0 *	3.3 ± 1.1	3.5 ± 0.9 *
Sex (male %)	0.7	0.6 *	0.4 *	0.9 *
Education (Year)	1.7 ± 1.2	1.4 ± 1.1 *	1.4 ± 1.2 *	1.4 ± 1.0 *
*Children characteristics*				
Age (Year)	10.3 ± 4.1	9.8 ± 3.7	9.6 ± 3.5	10.1 ± 4.1
Sex (male %)	0.6	0.6	0.6	0.6
BMI (Kg/m^2^)	17.4 ± 3.3	16.8 ± 3.0 *	16.9 ± 3.2	16.6 ± 2.7 *
Weight (Kg)	35.4 ± 15.2	31.6 ± 13.5 *	31.5 ± 13.7 *	31.8 ± 13.2
Height (cm)	138.9 ± 22.5	134.2 ± 21.2 *	133.5 ± 20.9 *	135.4 ± 21.8
Time gap	3.2 ± 1.7	3.1 ± 1.6	3.0 ± 1.6	3.1 ± 1.6

**^a^** all values represented as mean ± S.D, the median of income is also presented below the mean; **^b^** child_ratio: number of children in one family divided by the number of persons in this family; * compared with non-left children; significant level is set as *p* value < 0.016, which was corrected by Bonferroni correction 0.05/3 = 0.016.

**Table 2:** Comparison of growth indices for different children.

Indices	Non-Left	Left-Behind	Father-Left	At Least Mother-Left
*Follow-up*				
BMI	17.9 ± 3.6 **^a^**	17.2 ± 3.3	17.4 ± 3.6	16.7 ± 2.8 *
Weight	40.0 ± 15.3	35.7 ± 13.1 *	35.7 ± 13.7	35.7 ± 12.5
Height	146.2 ± 19.5	141.9 ± 17.7	140.8 ± 17.6	143.7 ± 17.9
*Baseline*				
BMI	16.9 ± 2.8	16.4 ± 2.6	16.3 ± 2.6	16.4 ± 2.7
Weight	30.7 ± 13.6	27.5 ± 12.6	27.3 ± 12.5	27.9 ± 13.0
Height	131.5 ± 22.8	126.5 ± 21.7	126.2 ± 21.4	127.2 ± 22.4
Normalized by the Chinese children growth standard				
*Follow-up*				
BMI_sd **^b^**	1.011 ± 0.179	0.986 ± 0.178	1.006 ± 0.204	0.955 ± 0.122 *
Weight_sd	0.995 ± 0.215	0.943 ± 0.233	0.950 ± 0.265	0.931 ± 0.169
Height_sd	1.032 ± 0.067	1.016 ± 0.066	1.010 ± 0.064 *	1.027 ± 0.068
*Baseline*				
BMI_sd	1.011 ± 0.149	0.994 ± 0.136	1.000 ± 0.143	0.986 ± 0.125
Weight_sd	0.986 ± 0.185	0.928 ± 0.188 *	0.939 ± 0.204	0.909 ± 0.158 *
Height_sd	1.020 ± 0.070	0.996 ± 0.072 *	0.998 ± 0.077	0.992 ± 0.063 *

**^a^** all values represented as mean ± S.D; **^b^** sd: means the value was normalized with the Chinese children growth standards; * *p* value < 0.016, which was corrected by Bonferroni correction 0.05/3 = 0.016.

**Table 3:** Average treatment effect of treated on growth caused by parents’ migration.

Indicator	Baseline				Follow-Up				ATT	
	Control	Treated	Difference	*p*	Control	Treated	Difference	*p*	DID ^b^	*p*
*Left-behind*										
BMI_sd **^a^**	1.011	0.994	−0.017	0.288	1.011	0.986	−0.025	0.223	−0.008	0.756
Weight_sd	0.986	0.928	−0.058	0.006	0.995	0.943	−0.052	0.046	0.006	0.846
Height_sd	1.020	0.996	−0.024	0.003	1.032	1.016	−0.016	0.037	0.008	0.415
*Father-left*										
BMI_sd	1.011	1.000	−0.011	0.563	1.011	1.006	−0.005	0.843	0.006	0.861
Weight_sd	0.986	0.939	−0.047	0.091	0.995	0.950	−0.045	0.209	0.002	0.962
Height_sd	1.020	0.998	−0.022	0.034	1.032	1.010	−0.022	0.013	0.000	0.982
*Mother-left*										
BMI_sd	1.011	0.986	−0.025	0.241	1.011	0.955	−0.056	0.011	−0.031	0.320
Weight_sd	0.986	0.909	−0.077	0.005	0.995	0.931	−0.064	0.031	0.013	0.735
Height_sd	1.020	0.992	−0.028	0.009	1.032	1.027	−0.005	0.656	0.023	0.142

**^a^** sd means the value was normalized with the Chinese children growth standards; **^b^** DID was calculated as: difference in follow-up minus difference in baseline.

**Table 4:** Average treatment effect of treated (ATT) on nutrition caused by parents’ migration.

7.5	Baseline				Follow-Up				ATT	
	Control	Treated	Difference	*p*	Control	Treated	Difference	*p*	DID ^b^	*p*
*Left-behind*										
Calorie_sd **^a^**	0.686	0.723	0.037	0.256	0.630	0.590	−0.040	0.083	−0.077	0.052
Protein_sd	1.033	1.020	−0.013	0.788	0.915	0.830	−0.085	0.024	−0.072	0.224
Fat_sd	0.438	0.374	−0.064	0.028	0.435	0.371	−0.064	0.029	0.000	0.999
Carbohydrate_sd	1.678	1.853	0.175	0.068	1.702	1.612	−0.090	0.238	−0.265	0.031
*Father-left*										
Calorie_sd	0.686	0.749	0.063	0.076	0.630	0.582	−0.048	0.082	-0.111	0.014
Protein_sd	1.033	1.051	0.018	0.726	0.915	0.821	−0.094	0.038	−0.112	0.103
Fat_sd	0.438	0.385	−0.053	0.131	0.435	0.385	−0.050	0.170	0.003	0.952
Carbohydrate_sd	1.678	1.939	0.261	0.036	1.702	1.569	−0.133	0.109	−0.394	0.008
*Mother-left*										
Calorie_sd	0.686	0.679	−0.008	0.889	0.630	0.602	−0.028	0.409	−0.021	0.749
Protein_sd	1.033	0.970	−0.063	0.421	0.915	0.845	−0.071	0.194	−0.008	0.934
Fat_sd	0.438	0.356	−0.081	0.056	0.435	0.348	−0.086	0.021	−0.005	0.929
Carbohydrate_sd	1.678	1.712	0.033	0.794	1.702	1.683	−0.018	0.881	−0.052	0.770

**^a^** sd refers to standardized using age-gender specific reference provided by Chinese dietary reference intake handbook; **^b^** DID was calculated as: difference in follow-up minus difference in baseline.

## Supplementary Materials

The following are available online at www.mdpi.com/1660-4601/14/11/1308/s1, Table S1: Distribution of individuals over four waves, Table S2: Comparison of characteristics between selected and excluded samples, Table S3: Chinese children growth standards, Table S4: Average treatment effect of treated on growth caused by parents’ migration-PSM-DID, Table S5: Average treatment effect of treated on nutrition caused by parents’ migration-PSM-DID.
