# Supplementary material for: Does Parental Migration Have Negative Impact on the Growth of Left-Behind Children?—New Evidence from Longitudinal Data in Rural China
Source: Int J Environ Res Public Health. 2017 Oct 27;14(11):1308. doi: 10.3390/ijerph14111308 (PMC5707947; doi:10.3390/ijerph14111308)
Supplement: Supplementary file 1 [file ijerph-14-01308-s001.pdf]

Does Parental Migration Have Negative Impact on the Growth of Left-Behind Children?—New Evidence from Longitudinal Data in Rural China

Xu Tian [1], Caicui Ding [2], Chong Shen [3] and Hui Wang [3,*]

The CHNS data

The CHNS is an international collaborative project between the Carolina Population Centre at the University of North Carolina at Chapel Hill and the National Institute for Nutrition and Health at the Chinese Centre for Disease Control and Prevention. The CHNS is an ongoing cohort survey on approximately 4000 families each year with a range of surveys covering the urban and rural areas in the following nine provinces (regions) before 2011: Guangxi, Guizhou, Henan, Heilongjiang, Hubei, Hunan, Jiangsu, Liaoning, and Shandong. The three municipalities of Beijing, Chongqing, and Shanghai were included in 2011. The content of the survey comprised the socioeconomic conditions, health services, residents' diet structures, and their nutritional statuses (Zhang et al., 2014; Wang et al., 2016).

A multistage, random cluster process was used to draw the sample surveyed in each of the provinces. Counties in the nine provinces were stratified by income (low, middle, and high) and a weighted sampling scheme was used to randomly select four counties in each province. In addition, the provincial capital and a lower income city were selected when feasible, except that other large cities rather than provincial capitals had to be selected in two provinces. Villages and townships within the counties and urban/suburban neighborhoods within the cities were selected randomly. From 1989 to 1993 there were 190 primary sampling units: 32 urban neighborhoods, 30 suburban neighborhoods, 32 towns (county capital city), and 96 rural villages. Since 2000, the primary sampling units have increased to 216: 36 urban neighborhoods, 36 suburban neighborhoods, 36 towns, and 108 villages (please see http://www.cpc.unc.edu/projects/china/about/design/survey).

Table S1. Distribution of individuals over four waves.

Baseline\Follow-Up	2006	2009	2011
2004	120	34	11
2006		95	29
2009			157

Note: row refers to the baseline period, and column refers to the follow-up period. Value is the number of individuals.

Table S2. Comparison of characteristics between selected and excluded samples.

Variable	Excluded (*n* = 1928)	Selected(*n* = 892)
Household characteristics		
Income (Yuan/year)	28258 ± 37744	28220 ± 34136
Income (median)	18371	19374
Household Size	5.0 ± 1.9	4.8 ± 2.0 *
Child ratio	0.3 ± 0.1	0.4 ± 0.1 *
Household head's characteristics		
Age (Year)	46.0 ± 13.9	44.8 ± 12.6 *
Activity	3.0 ± 1.2	3.1 ± 1.1 *
Sex (male%)	0.6	0.7 *
Education (Year)	1.6 ± 1.2	1.7 ± 1.1
Children characteristics		
Age (Year)	10.1 ± 4.6	10.2 ± 4.0
Sex (male %)	0.5	0.6
BMI ($Kg/m^{2)}$	17.7 ± 3.5	17.3 ± 3.2 *
Weight (Kg)	34.6 ± 15.6	34.5 ± 14.9
Height (cm)	136.5 ± 24.8	137.8 ± 22.3

Note: All values represented as mean ± S.D, the median of income is also presented below the mean; child_ratio is the number of children in one family divided by the number of persons in this family; * compared with excluded children; significant level is set as p <0.05.

Table S3. Chinese children growth standards.

Age	Weight_Boy	Height_Boy	Weight_Girl	Height_Girl	BMI_Boy	BMI_Girl
0	3.32	50.4	3.21	49.7	13.1	13
0.5	8.41	68.4	7.77	66.8	18	17.4
1	10.05	76.5	9.4	75	17.2	16.7
1.5	11.29	82.7	10.65	81.5	16.5	16
2	12.54	88.5	11.92	87.2	16.3	15.9
2.5	13.64	93.3	13.05	92.1	16	15.6
3	14.65	96.8	14.13	95.6	15.7	15.4
3.5	15.63	100.6	15.16	99.4	15.5	15.3
4	16.64	104.1	16.17	103.1	15.3	15.2
4.5	17.75	107.7	17.22	106.7	15.2	15.1
5	18.98	111.3	18.26	110.2	15.2	15
5.5	20.18	114.7	19.33	113.5	15.3	15
6	21.26	117.7	20.37	116.6	15.3	15
6.5	22.45	120.7	21.44	119.4	15.5	15
7	24.06	124	22.64	122.5	15.6	15
7.5	25.72	117.4	23.93	125.6	15.8	15.1
8	27.33	119.9	25.25	128.5	16	15.2
8.5	28.91	122.3	26.67	131.3	16.2	15.4
9	30.46	124.6	28.19	134.1	16.4	15.6
9.5	32.09	126.7	29.87	137	16.7	15.8
10	33.74	128.7	31.67	140.1	17	16.1
10.5	35.58	130.7	33.8	143.3	17.2	16.4
11	37.69	132.9	36.1	146.6	17.5	16.7
11.5	39.98	135.3	38.4	149.7	17.8	17.1
12	42.49	138.1	40.77	152.4	18.1	17.4
12.5	45.13	141.1	42.89	154.6	18.4	17.8
13	48.08	145	44.79	156.3	18.7	18.1
13.5	50.85	148.8	46.42	157.6	18.9	18.5
14	53.37	152.3	47.83	158.6	19.2	18.8
14.5	55.43	155.3	48.97	159.4	19.4	19.1
15	57.08	157.5	49.82	159.8	19.7	19.3
15.5	58.39	159.1	50.45	160.1	19.9	19.5
16	59.35	159.9	50.81	160.1	20.1	19.7
16.5	60.12	160.5	51.07	160.2	20.3	19.9
17	60.68	160.9	51.2	160.3	20.5	20
17.5	61.1	161.1	51.31	160.5	20.7	20.2
18	61.4	161.3	51.41	160.6	20.8	20.3

Note: The growth reference was based on a nine-city pilot study in China for various ages and sexes, which is conducted by Li et al. (2009).

Table S4. Average treatment effect of treated on growth caused by parents' migration-PSM-DID.

Indicator	Baseline				Follow-Up				ATT	
	Control	Treated	Difference	P	Control	Treated	Difference	P	DID	P
Left-behind										
BMI_sd	1.004	0.994	−0.010	0.532	1.003	0.986	−0.016	0.415	−0.007	0.794
Weight_sd	0.975	0.928	−0.047	0.027	0.986	0.943	−0.043	0.105	0.005	0.893
Height_sd	1.018	0.996	−0.022	0.008	1.031	1.016	−0.015	0.051	0.007	0.524
Father-left										
BMI_sd	1.001	1.008	0.007	0.728	0.995	1.013	0.018	0.515	0.011	0.737
Weight_sd	0.973	0.947	−0.025	0.365	0.979	0.957	−0.022	0.539	0.003	0.950
Height_sd	1.017	0.999	−0.018	0.092	1.031	1.011	−0.020	0.031	−0.002	0.913
Mother-left										
BMI_sd	1.006	0.987	−0.020	0.382	1.007	0.955	−0.052	0.018	−0.032	0.304
Weight_sd	0.976	0.908	−0.068	0.016	0.988	0.931	−0.057	0.056	0.011	0.783
Height_sd	1.018	0.992	−0.026	0.018	1.031	1.027	−0.004	0.715	0.022	0.170

Note: Results were estimated using the kernel-based propensity score matching (PSM) DID. Covariates used in estimation including time gap between two periods, household characteristics (net income, household size, ratio of children in the household, children characteristics (age and gender), and characteristics of household head (age, gender, education level, and physical activity).

Table S5. Average treatment effect of treated on nutrition caused by parents' migration-PSM-DID.

Indicator	Baseline				Follow-Up				ATT	
	Control	Treated	Difference	P	Control	Treated	Difference	P	DID	P
Left-behind										
Calorie_sd	0.683	0.723	0.039	0.217	0.639	0.590	−0.050	0.044	−0.089	0.027
Protein_sd	1.020	1.020	−0.000	0.999	0.924	0.830	−0.094	0.016	−0.094	0.123
Fat_sd	0.425	0.374	−0.051	0.087	0.434	0.371	−0.063	0.038	−0.012	0.776
Carbohydrate_sd	1.675	1.853	0.178	0.061	1.732	1.612	−0.120	0.138	−0.298	0.017
Father-left										
Calorie_sd	0.681	0.754	0.073	0.047	0.634	0.585	−0.049	0.090	−0.122	0.009
Protein_sd	1.022	1.056	0.035	0.520	0.917	0.831	−0.087	0.066	−0.121	0.091
Fat_sd	0.427	0.380	−0.047	0.204	0.432	0.384	−0.048	0.210	−0.001	0.983
Carbohydrate_sd	1.650	1.957	0.307	0.015	1.698	1.577	−0.121	0.162	−0.427	0.005
Mother-left										
Calorie_sd	0.686	0.666	−0.020	0.716	0.637	0.605	−0.033	0.378	−0.012	0.851
Protein_sd	1.020	0.950	−0.070	0.370	0.918	0.845	−0.073	0.205	0.004	0.970
Fat_sd	0.410	0.362	−0.048	0.268	0.435	0.354	−0.080	0.048	−0.032	0.583
Carbohydrate_sd	1.731	1.671	−0.060	0.631	1.754	1.688	−0.066	0.619	−0.006	0.976

Note: Results were estimated using the kernel-based propensity score matching (PSM) DID. Covariates used in estimation including time gap between two periods, household characteristics (net income, household size, ratio of children in the household, children characteristics (age and gender), and characteristics of household head (age, gender, education level, and physical activity).

© 2016 by the authors; licensee MDPI, Basel, Switzerland. This article is an open access article distributed under the terms and conditions of the Creative Commons by Attribution (CC-BY) license (http://creativecommons.org/licenses/by/4.0/).
